# The Effects of Cognitive Control on the Subcomponents of Language Control in Spoken and Written Productions

**DOI:** 10.3390/bs13100809

**Published:** 2023-09-29

**Authors:** Tingting Yang, Weihao Lin, Guorui Zheng, Ruiming Wang

**Affiliations:** Philosophy and Social Science Laboratory of Reading and Development in Children and Adolescents, Ministry of Education, & Centre for Studies of Psychological Application, School of Psychology, South China Normal University, Guangzhou 510631, China

**Keywords:** spoken production, written production, mixing costs, Simon task, AX-CPT task

## Abstract

Aims: The present study aimed to investigate whether and how the subcomponents of language control during spoken and written productions were modulated by cognitive control. Method: In the current study, unbalanced Chinese–English bilinguals were recruited from a convenience sample at a university to complete the cued language naming task in spoken production and written production, which measured the local language control (as indexed by language switch costs) and the global language control (as indexed by language mixing costs and reversed language dominance effect). In addition, all the participants performed the Simon task, which measured their general inhibitory control ability by calculating the Simon effect, and performed the AX-CPT task to measure their reactive/proactive control preference by calculating their BSI score. All the data were collected using E-prime 2.0 and analyzed in R. Linear mixed-effect model analyses were conducted to reveal the similarities and differences between spoken production and written production for the first-step analysis. Then, the Simon effect and BSI scores were inserted into the mixed-effect models of the switch costs and mixing costs in spoken production and written production, respectively, to explore whether cognitive control can predict the subcomponents of bilingual control. Results: The results showed similar symmetrical switch costs in spoken and written modalities. In contrast, there was a reversed language dominance effect (in the mixed language context) and asymmetrical mixing costs in spoken production but neither in written production. Furthermore, we found that the Simon effect significantly negatively predicted the L2 mixing costs in spoken production, whereas the BSI score significantly negatively predicted both the L1 and L2 mixing costs in written production. Conclusion: The findings indicated that, for unbalanced bilinguals, local language control is shared between two modalities, while global language control is modality-independent between spoken production and written production. More importantly, the findings also suggested that global language control in spoken production relies more on the individuals’ general inhibitory control, while in written production, it relies more on their cognitive control strategy. Global language control in spoken and written productions separately engages specific aspects of cognitive control, which may account for different forms of processing in global language control between speaking and writing.

## 1. Introduction

There is significant evidence that target and the non-target languages are both activated in bilingual language production [1,2,3], and that the non-target language interferes with the selection of and access to the target language [4,5]. Therefore, language control mechanisms are crucial for bilinguals to detect and resolve cross-language interference.

Bilingual language control is commonly investigated using the cued language switching paradigm. In such a task, bilinguals name pictures or digits in their native language (L1) or second language (L2) according to a given language cue (e.g., a flag or a color frame) in single language blocks (naming in either L1 or L2 in two separate blocks) and mixed language blocks (alternately naming the picture in L1 and L2). For mixed-language blocks, the naming language of two sequential trials can be the same (repeat trials) or different (switch trials). Typically, bilinguals perform with slower naming speeds and make more mistakes on switch trials than on repeat trials, which is known as the language switch cost [6,7,8]. Another important phenomenon is that some studies observed worse performance in L1 than in L2 under mixed language blocks. This is referred to as the reversed language dominance effect (e.g., [1,9,10,11,12]). In addition, their performance during repeat trials with mixed language blocks was worse than in trials with single language blocks, which is called the language mixing cost [9,13,14,15].

Interestingly, language switch costs are often asymmetrical in unbalanced bilinguals; that is, there are larger L1 switch costs compared to L2 switch costs [5,11,16]. Additionally, numerous studies have observed that language mixing costs are also asymmetrical across languages for unbalanced bilinguals; that is, there are larger mixing costs in L1 than in L2 (e.g., [14,15,16,17,18]). Many researchers have argued that these phenomena (including asymmetry in language switch costs or language mixing costs and the aforementioned reversed language dominance effect) reflect different types of language control modes (e.g., [9,16,18,19]). That is, asymmetrical language switch costs have been used as indicators of local or transient language control, which refers to the language control process that is engaged when the non-target language interferes with the selection of target language words. This is more transient and local (trial-by-trial) in nature. The reversed language dominance effect and asymmetrical language mixing cost have been assumed to be the indicators of global or sustained language control for bilinguals, which is reflected in implementation as anticipation of the selection of target words. It is a preventive control process. For both models, however, it is assumed that language control is primarily achieved through the inhibition of one language (i.e., inhibitory control; [4,20,21]).

According to the inhibitory control model (IC model, see [4,5]), bilinguals control their languages by inhibiting the activation level of the non-target language while accessing lexical representations of the target language. The asymmetrical switch costs suggest that unbalanced bilinguals inhibit their L1 during L2 speaking to a greater extent than inhibiting their L2 during L1 speaking. Consequently, bilinguals encounter greater processing difficulties when they reactivate a more heavily inhibited L1 when switching from L2 to L1 than when they reactivate a less heavily inhibited L2 when switching from L1 to L2 [22,23,24]. The asymmetric mixing cost, on the one hand, might be due to stronger sustained or global inhibition of the dominant L1 than the non-dominant L2 in a bilingual context [9,14]. In accordance with this view, some studies have reported worse L1 than L2 performance in the mixed language context [13,17,23,24]. On the other hand, because the baseline activation of L1 is higher than that of L2 for unbalanced bilinguals, L1 naming is usually faster and more accurate than L2 naming in a single language context [25,26]. Therefore, unbalanced bilinguals may engage in stronger global control in L1 than in L2.

Nevertheless, the empirical results on the above three phenomena are mixed. Although many studies have observed (one or more of) these effects (i.e., [3,5,13,27]), many studies have not reported any of these effects [28]. The decreased robustness of these effects may be due to the fact that they were modulated by language proficiency (e.g., [1,5]), language contexts (e.g., alternation context versus dense code switch context; [29]), preparation time (e.g., [16,30,31]), types and numbers of stimuli [30,32], and individual general cognitive control (e.g., [22,33]). Collectively, these studies suggest that linguistic task-related parameters and cognitive abilities may be key factors in revealing the cognitive nature of bilingual language control.

Notably, to investigate bilingual language control, most studies have focused on spoken production, while few studies have focused on written production. Recent research has extended the switching paradigm to written production and compared it to spoken production [34,35]. Wong and Maurer [34] conducted the first empirical study to explore whether switch costs existed in written production. In this study, they applied a sequence-based language switching paradigm and found no difference in switch costs between spoken and written modalities, indicating that some aspects of bilingual language control might be similar in spoken and written productions. In addition, another study observed similar asymmetrical patterns of switch costs when the response-to-stimulus interval was short both in spoken and written modalities, whereas the mixing costs were asymmetrical in spoken production and symmetrical in written production [35]. These findings indicate that there were similar mechanisms of local language control, but specific mechanisms of global language control between spoken and written production. Regrettably, these studies did not delve into the underlying causes to account for the different global language control mechanisms between the two language production modalities.

To our knowledge, there has been a multitude of studies providing crucial evidence demonstrating the overlapping mechanisms between cognitive control and language control in bilingual production [14,36,37,38]. On the one hand, some studies have observed that the general inhibitory control capacity of bilinguals has a certain impact on bilingual language control [22,33,39]. The Simon and Flanker tasks have been extensively used to measure individuals’ general inhibitory control capacity, with a smaller interference effect (i.e., a smaller difference between incongruent conditions and congruent conditions), indicating a better inhibitory control capacity [40,41,42]. For instance, Liu et al. [33] recruited unbalanced bilinguals with different capacities of inhibitory control (high IC group vs. Low IC group) to perform a language-switching task. They found asymmetrical switch costs in the high IC group and symmetrical switch costs in the low IC group. In another study, Liu et al. [22] observed smaller switch costs in the high IC group compared to the low IC group. In addition, a number of behavioral correlational studies have found that bilinguals’ inhibitory control capacity (measured using the Simon task or Flanker task) predicted the language switch cost [14,36,38], language mixing cost [14,38], or the reversed language dominance effect [43]. These findings indicate that domain-general inhibition extensively engages in local language control and global language control in bilingual spoken production.

On the other hand, some studies have reported an association between the cognitive control strategy and bilingualism. The AX-continuous performance task (AX-CPT) has been extensively used to measure individual biases for proactive and reactive control. The dual mechanisms of control framework (DMC) posited that cognitive control operates through two sub-components [44,45]: the proactive control and reactive control modes. The proactive control mode reflects an anticipatory process, allowing individuals to anticipate and prepare for potential interference before it occurs. In contrast, the reactive control mode reflects a late correction process that takes effect when interference occurs after it. In bilingual research, numerous studies have compared monolinguals to highly proficient bilinguals or bilinguals with different proficiency levels using the AX-CPT task and found that bilingual experience can modulate cognitive control strategies [46,47,48,49].

Notably, a recent study recruited trilingual speakers to perform a modified language-switching task while simultaneously recording EEG signals, and to additionally complete an AX-CPT task [50]. They found that the cue stage elicited the late positive component (LPC) in the language task was negatively correlated with proactive control preference. Furthermore, another study explored how different bilingual experiences mediate the relationship between cognitive strategies and language control [51] and found that an integrated bilingual context (in which individuals use their two languages frequently and live in a predominantly L1 environment) with greater reliance on reactive control processes predicted higher language naming accuracy in both L1 and L2. Taken together, the existing evidence suggests a connection between cognitive control (including general inhibitory ability and cognitive control strategy) and language control performance. Hence, we try to adopt a correlational approach to reveal bilingual language control in spoken production and written production, whether associated with cognitive control.

In summary, the results pertaining to the three indicators related to bilingual control exhibit inconsistency across studies. Furthermore, limited evidence suggests that there are similar local language control mechanisms and different global language control mechanisms between spoken and written productions, and the reproducibility of this finding remains to be verified. Notably, while cognitive control and bilingual control in spoken production have been demonstrated to be closely interconnected, whether cognitive control is associated with bilingual control in written production is unknown. Therefore, whether there is a specific association between cognitive control and bilingual control in different language production modalities requires further research.

Responding to the above research gaps, the present study aims to investigate whether individual variability in cognitive control modulates the subcomponents of language control (local language control and global language control) and whether these modulations translate into differences in performance between spoken production and written production. To this end, we recruited unbalanced bilinguals to perform two cued language switching tasks and two cognitive control tasks (Simon task and AX-CPT task). The Simon task is a widely used task for measuring general inhibitory control ability in which the smaller Simon effect (i.e., the smaller difference between the incongruent condition and congruent condition) indicates a stronger inhibitory control ability [36,42,52]. The AX-CPT task measures proactive/reactive control strategies by calculating the behavioral shift index (BSI score). A higher BSI score indicates individual bias to engage in a proactive control strategy, and a lower BSI score indicates bias in a reactive control strategy [44,45,50]. The cued language switching tasks, which included separate spoken naming and written naming, were used to measure subcomponents of language control in the participants. Specifically, we attempted to reveal local language control processing indexed by language switch costs between spoken production and written production and global language control processing indexed by a reversed language dominance effect and language mixing costs between the two modalities. The Simon effect was calculated using the Simon task to measure the participants’ general inhibitory control abilities, and the behavioral shift index (BSI) was calculated using the AX-CPT paradigm to reflect the participants’ cognitive control strategy in the proactive or reactive state.

The first aim of this research was to find the similarities and differences in language control (local language control and global language control) between spoken and written tasks. Based on Yang et al. ’s study [35], we preliminarily predicted that both spoken and written production would exhibit similar processing mechanisms in local language control but dissimilar processing mechanisms in global language control. The second aim was to determine whether and how the relationship between individual cognitive control ability and bilingual language control performance is modulated by the types of production modalities. If global language control between spoken and written modalities is modulated differently through cognitive control capacity, this will provide an explanation for the performance differences between spoken and written production.

## 2. Methods

This study employed an experimental research design to explore the similarities and differences in the subcomponents of bilingual control between spoken and written productions, and to reveal their potential modulation by cognitive control in-depth. Firstly, we utilized a cued language naming task to assess bilingual control in both the spoken and written modalities. This task was selected to quantify the local language control (indexed by language switch costs) and global language control (indexed by language mixing costs and the reversed language dominance effect). In addition, we incorporated the Simon task to measure the participants’ general inhibitory control ability, quantified by the calculation of the Simon effect. The AX-CPT task was employed to gauge the participants’ reactive/proactive control preference, and the BSI score served as a quantitative descriptor.

### 2.1. Participants

This study used convenience sampling to recruit participants through advertisements. A total of 62 Chinese–English bilinguals from the South China Normal University took part in the study (44 females, mean age = 20.66, *SD* = 1.90). All the participants were right-handed, had normal or corrected-to-normal vision, and had no history of language disorders. They all signed a written informed consent form before participating, and the Institutional Ethics Committee of South China Normal University approved the study. According to the sample size calculation in G*power 3.1.9 software [53], 23 participants were needed to reach a medium-size effect (*f* = 0.25) with an alpha error probability of 0.05 and a power of 95%. Therefore, the participants in this experiment were appropriate.

All the participants were native Chinese speakers with no immigration or overseas education experience. They self-assessed their proficiency in listening, speaking, reading, and writing in Chinese and English based on a 7-point scale (1 = not fluent at all, 7 = very fluent). In addition, they completed a lexTALE test [54] to evaluate their English vocabulary levels. Paired samples t-tests showed that the self-assessed scores were significantly higher for Chinese than for English listening (*t* = 18.59, *p* < 0.001), speaking (*t* = 17.01, *p* < 0.001), reading (*t* = 13.03, *p* < 0.001), and writing (*t* = 9.58, *p* < 0.001), suggesting that the participants were unbalanced bilinguals with a dominant L1. Detailed information about the participants’ characteristics is shown in Table 1.

### 2.2. Materials and Procedure

#### 2.2.1. Cued Language Switching Task

The cued language switching task is a commonly used paradigm in bilingual research to measure language control processes [30,55]. This task is highly regarded for its validity and reliability in capturing various aspects of bilingual control. Numerous studies [56,57] have consistently reported the task’s effectiveness in providing valuable insights into real-time language control processes. The cued language switching task’s robustness in measuring language control makes it a dependable tool for bilingualism research.

For the cued language-switching task in this study, the materials included twelve black-and-white pictures (line drawings) from the picture set of Zhang and Yang [58]. Each picture was provided with a pair of Chinese and English names (see Appendix A). The Chinese names of the pictures were monosyllabic Chinese names (e.g., “花” in Chinese), with an average stroke count of 6.1 (ranging from 3 to 10). The average number of letters in the pictures’ English names (e.g., “flower” in English) was 4.0 (ranging from 3 to 6). The same pictures were presented in the L1 single, L2 single, and mixed language blocks.

**Figure 1:**
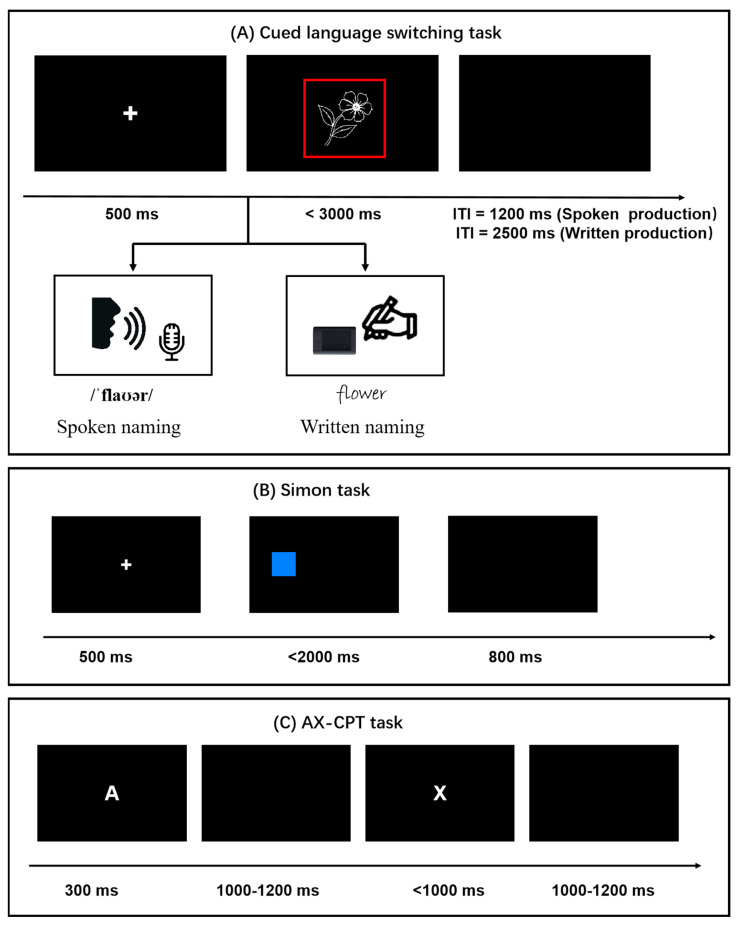
Illustration of the cued language switching tasks and cognitive control tasks in this study.

The participants were first familiarized with these pictures and their Chinese and English names via a PowerPoint presentation. Next, they performed the cue language-switching task separately in spoken and written modalities, which was run on E-Prime 2.0 on a desktop. The order of the response production was counterbalanced across the participants. The structure of the trials in the two modalities is illustrated in Figure 1A. A trial started with a fixation cross (+) in the center of the screen for 500 ms, followed by a picture framed inside a red or green square. The participants were asked to name the picture as rapidly and accurately as possible in a specific language depending on the color of the frame (specifically, for half of the participants, red indicated naming in Chinese and green indicated naming in English, while for the other half, it was the opposite). For instance, when the participants were presented with a black-and-white picture of a flower within a red (or green) square, they were required to speak (or write) the name of the flower in Chinese (“花”).

The participants responded to the picture naming using spoken or written modalities, and the picture disappeared once the participants initiated a spoken/written response or if they did not respond within 3 s. For the spoken naming responses, the naming latencies were digitally recorded using a voice key connected to the computer via a Chronos response box. The inter-trial interval was presented for 1200 ms, which reflects the interval from the spoken response onset to the presentation of the next fixation. In addition, we conducted a video recording of the spoken task through the video recording software EV Capture to check the accuracy of the spoken response. For the written naming responses, the naming latencies were digitally recorded using a WACOM Intuos A4 graphic tablet with a WACOM inking digitizer pen (Wacom, Japan). An inter-trial interval was presented for 2500 ms in the written responses. Afterward, we checked the accuracy of the written responses according to the handwriting on the paper.

Both the spoken and written naming tasks began with two monolingual blocks (one Chinese naming and one English naming with the naming language order balanced between the participants) with 36 trials each. Then, three mixed language blocks were conducted with 49 trials each. Every single-language block was preceded by 12 practice trials, and the first mixed block was preceded by 24 practice trials. The first trial in the mixed-language blocks was excluded from the analysis because it was neither a switch trial nor a repeat trial. Therefore, there were 36 trials for each trial type (single, repeat, and switch) within each language for both tasks.

#### 2.2.2. Simon Task

The Simon Task is a well-validated tool for assessing inhibitory control abilities in a range of cognitive domains, including bilingual research. Its effectiveness in measuring inhibitory control in bilingual contexts is supported by a body of literature [36,43,59].

For the Simon task used in this study, the structure of the trials was as follows (see Figure 1B): each trial started with a fixation cross (+) in the center of the screen for 500 ms, followed by a square (blue or orange) appearing on the left or right side of the computer screen with the same probability of occurrence for each position. The participants were asked to respond as soon as possible according to the color of the square by pressing a key on the right side of the keyboard for orange squares and a key on the left side of the keyboard for blue squares. The matching of the response keys and color squares was counterbalanced across the participants. When the location of the square and the correct response on the same side were deemed to be in a congruent condition, the location of the square and the correct response on the different sides were deemed to be in an incongruent condition. The Simon effect was calculated based on the difference between the congruent and incongruent conditions, with a larger value reflecting a weaker ability in general inhibitory control. In the present study, the Simon task consisted of a practice block (8 trials) and two formal blocks of 40 trials each. Thus, there were 40 formal trials in the congruent and incongruent conditions, respectively.

#### 2.2.3. AX-CPT Task

The AX-continuous performance task (AX-CPT task) is a widely recognized task for separating proactive and reactive control preferences, making it a valuable instrument in cognitive control research [44,51,60].

For the AX-CPT task used in this study, we applied a procedure similar to that used by Zhang et al. [61]. The structure of the trials was as follows (see Figure 1C): A trial started with a letter (either A or B) in the center of the screen for 300 ms, which appeared as a cue, followed by a blank screen for 1000–1200 ms. Then, the second letter (either X or Y) occurred as a target, which disappeared once the participants responded or when 2000 ms had elapsed. If the cue–target letter pair presented in the sequence was AX, the participants were instructed to press “D” using their left index finger, and if the sequence was AY, BX, or BY, the participants pressed “K” with their right index finger. Finally, an ITI was present for 1000–1200 ms until the next trial appeared. In total, the AX-CPT task consisted of a practice block (24 trials) and three formal blocks of 100 trials each. Thus, there was a total of 300 trials used for analysis for each participant, with the AX condition accounting for 70% of these trials. The other conditions (i.e., AY, BY, and BX) each occurred in 10% of the trials.

### 2.3. Data Analysis

#### 2.3.1. Cued Language Switching Task

In the cued language-switching task, for naming latencies, we first eliminated the trials that were not appropriately recorded due to voice-key triggering failure in spoken production and writing pen trigger failure in written production. Next, the error trials in which a wrong language or a wrong word (correct language) was used were discarded. Then, the trials with latencies below 300 ms or above 2500 ms were removed. Lastly, the trials with a latency beyond 2.5 SDs away from every participant’s mean were removed. Taking these criteria into account, 7.14% of the data were removed for spoken production, and 6.80% of the data were removed for written production. Considering that the distribution of the naming latencies was moderately skewed (skewness = 1.23), the latency data were log-transformed to reduce the skewness (skewness = 0.34 after transformation). Table 2 shows the mean latencies and error rates for the cued language switching tasks in the spoken and written modalities.

For the naming latency and error rate data, we utilized linear mixed-effects models (LME) (lmerTest package; [62]) to analyze the switch costs and mixing costs. The latency and error rate data were analyzed in sequence. For the switch costs, the modality (spoken vs. written), language (L1 vs. L2), trial type (switch vs. repeat), and their interactions were included in the model as fixed factors. Deviation contrasts (−0.5 and 0.5) were used for all the fixed effects so that the estimates for the factors reflected the main effects and interactions. For the mixing costs, the modality (spoken vs. written), language (L1 vs. L2), trial type (repeat vs. single), and their interactions were used as fixed factors. Similarly, deviation contrasts were also used for all the fixed effects. Post-hoc comparisons using the emmeans package [63]. The initial model included random intercepts for both the participants and items, as well as random slopes for modality, language, trial type, and their interaction for both the participants and items. The contribution of random slopes was evaluated through likelihood ratio testing, and the models were compared based on the Akaike information criterion (AIC) to determine the final model structure. The results for the best-fitting models that were verified by the data were reported.

#### 2.3.2. Simon Task

For the Simon task, trials with wrong responses, responses shorter than 100 ms or longer than 1000 ms, and response times 2.5 SDs above or below each participant’s mean value were sequentially removed from the response times analysis. Based on these criteria, 5.44% of the data were removed for the Simon task. For the behavioral performance under congruent and incongruent conditions, the reaction times were *M* = 423 ± 105 ms and *M* = 441 ± 93 ms, respectively, and the accuracies were *M* = 97.1% ± 3.3% and *M* = 97.9% ± 2.6%, respectively.

To address the research question of whether language control performance is predicted by an inhibitory ability in bilinguals, we inserted the Simon effect (the difference between the incongruent condition and the congruent condition) into the mixed-effects models of the switch costs and mixing costs in spoken and written modalities, respectively. Regarding the switch costs, both models had log latencies as the dependent variable and language (L1 vs. L2), trial type (switch vs. repeat), the Simon effect, and their interaction as fixed effects. Similarly, the models involving mixing costs included language (L1 vs. L2), trial type (repeat vs. single), the Simon effect, and their interaction as fixed effects. In these models, language and trial types were both coded with deviation contrasts (−0.5 and 0.5), and the Simon effect was centered and treated as a continuous predictor.

#### 2.3.3. AX-CPT Task

For the AX-CPT task, trials with incorrect responses, responses shorter than 100 ms or longer than 1000 ms, and response times 2.5 SDs above or below each participant’s mean value were sequentially removed from the response times analysis [46,64]. In total, 6.37% of the data were removed in the AX-CPT task. Regarding the behavioral performances under AX, AY, BX, and BY conditions, the reaction times were *M* = 397 ± 102 ms, *M* = 496 ± 102 ms, *M* = 335 ± 140 ms, and *M* = 334 ± 130 ms, respectively, and the accuracies were *M* = 99.4% ± 0.7%,* M* = 86.6% ± 10%,* M* = 96.5% ± 3.9%, and *M* = 99.7% ± 0.1%, respectively.

Following a study by Braver et al. [44], we measured the behavioral shift index [BSI: (AY − BX)/(AY + BX)] of RTs and error rates as behavioral measures for individual control strategies. The BSI score ranged from −1 to +1, and a higher BSI score signified a worse performance in the AY condition and a better performance in the BX condition, indicating a preference for using the proactive control strategy. In contrast, a lower BSI score signified a better performance in the BX condition but a poor performance in the AY condition, indicating a preference for using the reactive control strategy.

Similarly, we inserted the behavioral shift index (BSI score) into the mixed-effects models of the switch costs and mixing costs separately in spoken and written modalities. The model involving switch costs considered language (L1 vs. L2), trial type (switch vs. repeat), BSI, and their interactions as fixed effects. And the model involving mixing costs included language (L1 vs. L2), trial type (repeat vs. single), BSI, and their interactions as fixed effects. In these models, the language and trial type were both coded with deviation contrasts (−0.5 and 0.5), and the BSI score was centered and treated as the continuous predictor.

## 3. Results

### 3.1. Comparison of Bilingual Language Control between the Spoken and Written Modalities

#### 3.1.1. Switch Costs and Reversed Language Dominance Effect

As shown in Table 3, an analysis of the log latencies demonstrated a significant main effect of modality (*β* = 0.260, *SE* = 0.016, *t* = 16.12, *p* < 0.001), where naming in written production (*M* = 1067 ms) had longer latency than in spoken production (*M* = 820 ms). The main effects of language (*β* = 0.029, *SE* = 0.013, *t* = 2.27, *p* = 0.039) and trial type (*β* = 0.077, *SE* = 0.007, *t* = 11.35, *p* < 0.001) were significant as well. The naming latencies were longer in L1 than in L2 (*M* = 959 ms vs. *M* = 929 ms) and were longer in the switch trials than in the repeat trials (*M* = 982 ms vs. *M* = 907 ms). In addition, there was a significant interaction effect between the modality and language (*β* = −0.022, *SE* = 0.007, *t* = −3.20, *p* = 0.002), indicating that the reversed language dominant effect was presented in spoken production but not in written production. More specifically, the participants had longer latencies in L1 than in L2 in spoken production (*M* = 839 ms vs. *M* = 802 ms; *β* = −0.040, *SE* = 0.014, *z* = −2.99, *p* = 0.003), while there was no difference between L1 and L2 in written production (*M* = 1076 ms vs. *M* = 1057 ms; *β* = −0.018, *SE* = 0.013, *z* = −1.38, *p* = 0.168). The other two-way interaction effects were not significant (*ps* > 0.5). Although the three-way interaction among modality, language, and trial type was not significant (*β* = −0.018, *SE* = 0.011, *t* = −1.56, *p* = 0.119), in order to determine the relative magnitude of the switch costs between L1 and L2, we further analyzed the switch costs under each production modality. The result showed that the interaction between language and trial type was not significant in both modalities (spoken production: *β* = 0.009, *SE* = 0.008, *z* = 1.11, *p* = 0.268; written production: *β* = −0.009, *SE* = 0.008, *z* = −1.10, *p* = 0.272), indicating that there were similar patterns of symmetrical switch costs in both modalities.

An analysis of the error rates revealed a significant main effect of language (*β* = 0.331, *SE* = 0.096, *z* = 3.45, *p* < 0.001), with higher error rates in L1 naming than in L2 naming (*M* = 3.3% vs. *M* = 2.4%). The trial type was significant as well (*β* = 0.567, *SE* = 0.096, *z* = 5.90, *p* < 0.001), showing that the switch trials had higher error rates compared to the repeat trials (*M* = 3.6% vs. *M* = 2.2%). Moreover, there was a significant two-way interaction between the modality and language (*β* = 1.045, *SE* = 0.192, *z* = 5.45, *p* < 0.001). Further analysis revealed that the error rates were significantly higher in L1 naming relative to L2 naming for spoken production (*M* = 4.2% vs. *M* = 2.0%; *β* = 0.854, *SE* = 0.138, *z* = 6.19, *p* < 0.001). In contrast, the error rates in L1 naming were comparable with L2 naming for written production (*M* = 2.4% vs. *M* = 2.9%; *β* = −0.191, *SE* = 0.133, *z* = 1.44, *p* = 0.151). None of the other main effects or interactions were significant (*ps* > 0.1).

#### 3.1.2. Mixing Costs

The model results of the log latencies for mixing costs are shown in Table 3. The significant main effect of modality (*β* = 0.265, *SE* = 0.016, *t* = 16.21, *p* < 0.001) revealed longer naming latencies in written production compared to spoken production (*M* = 955 ms vs. *M* = 730 ms). There was a significant main effect of trial type (*β* = −0.143, *SE* = 0.008, *t* = −17.14, *p* < 0.001), suggesting that the naming latencies were longer in the repeat trials than in the single trials (*M* = 907 ms vs. *M* = 783 ms). Additionally, the interaction between language and trial type was significant (*β* = −0.037, *SE* = 0.008, *t* = −4.48, *p* < 0.001), indicating that the mixing costs were greater in L1 than in L2 overall (*M* = 142 ms vs. *M* = 112 ms). More importantly, the three-way interaction among modality, language, and trial type was also significant (*β* = 0.041, *SE* = 0.013, *t* = 3.11, *p* = 0.003). Further analysis revealed that there was significant two-way interaction between language and trial type in spoken production (*β* = −0.058, *SE* = 0.010, *z* = −5.98, *p* < 0.001) but not in written production (*β* = −0.016, *SE* = 0.011, *z* = −1.43, *p* = 0.152). That is to say, the L1 mixing costs were significantly greater than the L2 mixing costs in spoken production (i.e., asymmetrical mixing costs; *M* = 136 ms vs. *M* = 92 ms). In contrast, the mixing costs were comparable between L1 and L2 in written production (i.e., symmetrical mixing costs; *M* = 147 ms vs. *M* = 132 ms). To summarize, there were asymmetrical mixing costs in the spoken production and symmetrical mixing costs in the written production.

An analysis of error rates revealed that the main effect of trial type was significant (*β* = 2.086, *SE* = 0.218, *z* = 9.59, *p* < 0.001), with higher error rates for repeat trials (*M* = 2.2%) than for single trials (*M* = 0.1%). In addition, there was a significant interaction between the modality and language (*β* = 0.845, *SE* = 0.435, *z* = 1.94, *p* = 0.052). Further analysis revealed that there were significantly higher error rates in written production than in spoken production for L2 naming (*M* = 1.4% vs. *M* = 0.7%; *β* = 0.633, *SE* = 0.311, *z* = 2.03, *p* = 0.042), whereas there were no significant differences in error rates between the written production and spoken production for L1 naming (*M* = 1.0% vs. *M* = 1.8%; *β* = −0.212, *SE* = 0.367, *z* = −0.58, *p* = 0.563). The significant interaction effect between language and trial type (*β* = 0.919, *SE* = 0.435, *z* = 2.11, *p* = 0.035) indicated that, overall, the mixing cost was greater in L1 (*β* = 2.54, *SE* = 0.337, *z* = 7.55, *p* < 0.001) than in L2 (*β* = 1.63, *SE* = 0.275, *z* = 5.91, *p* < 0.001). None of the other effects were significant (*ps* > 0.1).

### 3.2. Bilingual Language Control and Cognitive Control Ability

#### 3.2.1. The Association between Language Control Performance and Individuals’ General Inhibitory Control Ability

For language control performance in spoken production, the analysis results involving language (L1 vs. L2), trial type (i.e., switch cost: switch vs. repeat), and the Simon effect are as follows: the interaction effect between language and the Simon effect was not significant (*β* = −0.133, *SE* = 0.097, *t* = −1.37, *p* = 0.177), indicating that the Simon effect did not predict the reversed language dominance effect. Also, the interaction effect between the trial type and the Simon effect separately under L1 baseline (*β* = −0.036, *SE* = 0.096, *t* = −0.37, *p* = 0.710) and L2 baseline (*β* = 0.142, *SE* = 0.095, *t* = 1.50, *p* = 0.135) did not reach significantly, indicating that the Simon effect did not predict the switch costs in L1 and L2. In addition, the analysis results involving language (L1 vs. L2), trial type (i.e., mixing cost: repeat vs. single), and the Simon effect are as follows: the significant interaction between language and the Simon effect (*β* = −0.342, *SE* = 0.106, *t* = −3.22, *p* = 0.002) suggested that the Simon effect negatively predicted the log latency differences across L1 and L2 when combined with the repeat trials and single trials. Further analysis demonstrated that interaction between language and the Simon effect was significant for the single trial baseline (*β* = 0.462, *SE* = 0.129, *t* = −3.59, *p* = 0.001) and marginally significant under the repeat trial baseline (*β* = 0.222, *SE* = 0.126, *t* = 1.77, *p* = 0.082). That is, the participants with a better inhibition capacity (i.e., lower Simon effect) tended to perform with greater naming speed differences across languages for single trials. More importantly, the Simon effect significantly predicted the mixing costs in L2 (*β* = −0.337, *SE* = 0.143, *t* = −2.35, *p* = 0.022) but not in L1 (*β* = −0.097, *SE* = 0.155, *t* = −0.63, *p* = 0.532). Specifically, participants with better inhibition capacities performed stronger global language control of their weaker L2.

**Figure 2:**
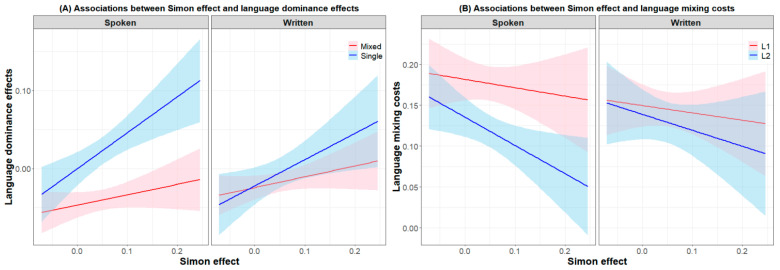
(**A**) Associations between the Simon effect and language dominance effects in spoken production and written production. (**B**) Associations between the Simon effect and language mixing costs in the two modalities.

For language control performance in written production, an analysis involving language, switch cost, and the Simon effect revealed that the Simon effect did not predict the reversed language dominance effect (*β* = 0.000, *SE* = 0.000, *t* = −1.48, *p* = 0.144), L1 switch cost (*β* = 0.000, *SE* = 0.000, *t* = −1.12, *p* = 0.267), or L2 switch cost (*β* = 0.000, *SE* = 0.000, *t* = 1.27, *p* = 0.209). An analysis involving language, mixing cost, and the Simon effect revealed that the Simon effect significantly negatively predicted the log latency differences across L2 and L1 when repeat trials and single trials in written production were combined (*β* = −0.287, *SE* = 0.106, *t* = −2.72, *p* = 0.009). Further analysis revealed a significant interaction between language and the Simon effect under the single trial baseline (*β* = 0.339, *SE* = 0.142, *t* = 2.39, *p* = 0.020) and a marginally significant interaction under the repeat trial baseline (*β* = 0.235, *SE* = 0.129, *t* = 1.82, *p* = 0.074). This finding was similar in the spoken production. As observed in Figure 2A, the lower the inhibitory control ability of the participants was, the greater the language dominance effect (i.e., log latency differences across L2 and L1) in the single-language context in both the spoken and written modalities. Compared to another finding in spoken production, the Simon effect did not predict the L1 mixing cost (*β* = −0.089, *SE* = 0.154, *t* = −0.57, *p* = 0.569) or L2 mixing cost (*β* = −0.193, *SE* = 0.182, *t* = −1.06, *p* = 0.292) in written production. As observed in Figure 2B, participants with a stronger inhibitory control ability (i.e., smaller Simon effect) tended to exhibit a greater L2 mixing cost in spoken production.

#### 3.2.2. Association between Language Control Performance and Individuals’ Proactive Control Strategies

For language control performance in spoken production, an analysis involving language, switch cost, and BSI score revealed that the BSI score significantly negatively predicted the reversed language dominance effect (*β* = −0.177, *SE* = 0.084, *t* = −2.11, *p* = 0.039). That is, the participants with stronger proactive preferences (i.e., higher BSI score) tended to perform with a smaller reversed language dominance effect. Additionally, the BSI score did not predict the switch costs in L1 (*β* = −0.009, *SE* = 0.085, *t* = −0.11, *p* = 0.915) or in L2 (*β* = −0.041, *SE* = 0.084, *t* = −0.49, *p* = 0.627). An analysis involving language, mixing cost, and BSI score demonstrated that the BSI score did not predict naming speed differences across the two languages (single trials: *β* = 0.102, *SE* = 0.125, *t* = 0.82, *p* = 0.418; repeat trials: *β* = 0.162, *SE* = 0.112, *t* = 1.45, *p* = 0.153), L1 mixing cost (*β* = −0.158, *SE* = 0.136, *t* = −1.16, *p* = 0.249), or L2 mixing cost (*β* = −0.097, *SE* = 0.132, *t* = −0.74, *p* = 0.463) in spoken production.

**Figure 3:**
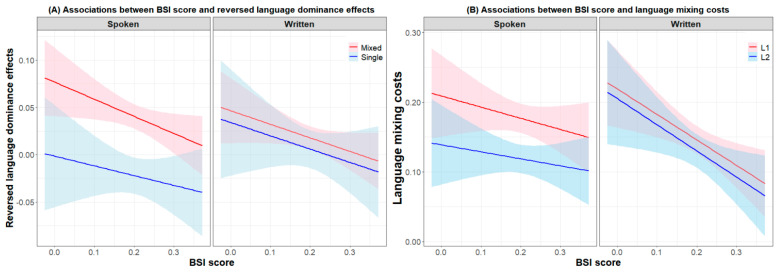
(**A**) Associations between BSI score and reversed language dominance effects in spoken production and written production. (**B**) Associations between BSI score and language mixing costs in the two modalities.

For language control performance in written production, an analysis involving language, switch cost, and BSI score revealed that the interaction between language and BSI was marginally significant (*β* = −0.140, *SE* = 0.079, *t* = −1.78, *p* = 0.081), suggesting that participants with a stronger proactive preference (i.e., higher BSI score) tended to perform with a smaller reversed language dominance effect in written production, similar to the results for spoken production. The L1 switch costs (*β* = −0.062, *SE* = 0.094, *t* = −0.66, *p* = 0.510) and the L2 switch cost (*β* = −0.067, *SE* = 0.090, *t* = −0.74, *p* = 0.460) in written production were not predicted by the BSI score. An analysis involving language, mixing cost, and BSI score demonstrated that the naming speed differences across two languages were not predicted by the BSI score (single trials: *β* = 0.140, *SE* = 0.130, *t* = 1.07, *p* = 0.287; repeat trials: *β* = 0.136, *SE* = 0.116, *t* = 1.17, *p* = 0.246). As observed in Figure 3A, the stronger the proactive control preference of the participants was, the smaller the reversed language dominance effect (i.e., log latency differences across L1 and L2) in the mixed-language context in both spoken and written modalities. Contrary to the findings for spoken production, the BSI score significantly negatively predicted the L1 mixing cost (*β* = −0.370, *SE* = 0.128, *t* = −2.88, *p* = 0.005) and the L2 mixing cost (*β* = −0.373, *SE* = 0.155, *t* = −2.41, *p* = 0.019) in written production. As observed in Figure 3B, participants with a stronger proactive control preference tended to perform with smaller mixing costs in written production.

## 4. Discussion

The present study investigated whether there were general and specific bilingual control mechanisms between spoken production and written production, and whether and how the individual variability in cognitive control modulated the different types of language control in spoken and written production. The results revealed that, in a cued language switching paradigm, bilinguals exhibited similar local language control in spoken and written modalities, but with different patterns of global language control between the two modalities. Importantly, by combining the language control performance and cognitive factors in tandem, this study gives insight into why global language control differs between the two production modalities. Our findings suggest that individuals’ inhibitory control engages in the L2 global language control in spoken production, whereas individuals’ proactive control preference engages in the L1 and L2 global language control in written production. We discuss the implications of our findings in the following section.

### 4.1. Similarities and Differences in Control Mechanisms between Bilingual Speaking and Writing 

Our first research aim was concerned with the similarities and differences in bilingual language control between spoken production and written production. On the one hand, the present study found a similar pattern regarding language switch costs in the two modalities. Specifically, both spoken and written modalities showed symmetrical switch costs, with switch costs comparable between L1 and L2. This finding is inconsistent with a recent study that observed asymmetrical switch costs in short response–stimulus interval (RSI) conditions both in spoken and written modalities [35]. In Yang et al.’s study [35], they found that the asymmetry between the switch costs was modulated by the RSI length, with asymmetrical switch costs appearing in shorter RSIs which were decreased or even absent during longer RSIs. A similar modulation effect of RSI was also reported by Ma et al. [16] in spoken production.

A potential explanation for this symmetry or asymmetry in switch costs is that the repetition and the cumulative number of trials may also be factors affecting bilingual language control. Kleinman and Gollan [32] re-analyzed data from 416 Spanish–English bilinguals and found that bilingual language control is not only affected by the order of blocks, but also by the number of trials in a block. Therefore, the activation states across language types and trial types continuously change over time during increasingly cumulatively longer periods of mixed language. In this study, they observed that the switch costs for the dominant language were greater than for the non-dominant language in block quarters 1, 3, and 4. The exception to this tendency was in block quarter 2, with undifferentiated switch costs across these two languages. In fact, the number of trials per condition (36 trials) in the present study was lower than the 48 trials per condition in Yang et al.’s study. This symmetrical switch cost in this study aligns with the results of spoken production for unbalanced bilinguals in the study by Ivanova and Hernandez [65], who had the same trial number per condition as in this study. Therefore, we speculate that the difference in the number of trials in each condition may be responsible for the discrepancy between the results of this study and those of Yang et al.

In addition, a recent meta-analysis by Gade et al. [66] could not find clear evidence for an asymmetry in switch cost when systematically assessing the evidence (i.e., independent of citation biases). That is, for unbalanced bilinguals, there are approximately as many studies that showed asymmetries in switch costs (e.g., [16,17,18]) as there were studies that showed symmetries in switch costs (e.g., [12,13,65]).

The most likely explanation is that the local language control mechanism is dynamic and may exhibit varying degrees of cross-language competition, depending on a host of factors such as the task paradigm (e.g., voluntary language switching versus language switching; [67]), language contexts (e.g., alternation context versus dense code switch context; [29]), cue–stimulus interval [16,31], response–cue intervals [16,35], types and numbers of stimuli [32], individual language proficiency (e.g., [1,5]), and individual general cognitive control abilities (e.g., [22,33]).

When controlling for multiple influencing factors, spoken and written production consistently exhibited similar processing patterns in local language control, either both with stronger local language control in the dominant L1 (Yang et al. [35]) or both with similar local language control across L1 and L2 (this study). Taken together, these studies commonly support that local language control was shared between spoken production and written production (i.e., present study; [34,35]).

On the other hand, in the present study, different patterns of language dominance effects and language mixing costs were both found between the two modalities. The reversed language dominance effects and language mixing costs were used as indicators of global language control, reflecting global inhibitory processing for cross-language interference [9,16]. In the mixed-language context, there was a reversed language dominance effect in spoken production, such that the dominant L1 exhibited slower naming latencies and higher error rates than the non-dominant L2. In contrast, the behavioral performance in L1 was comparable with L2 in written production. In addition, the mixing cost was asymmetrical across L1 and L2 in spoken production but was symmetrical in written production. This asymmetrical pattern of mixing cost is consistent with numerous studies on bilingual spoken production (e.g., [13,14,18,65]), where the mixing cost is greater in the dominant L1 compared to non-dominant L2 for unbalanced bilinguals. Of note, these findings in global language control are in line with the result of Yang et al., who revealed a reversed language dominance effect and asymmetrical mixing cost in spoken production but not in written production by comparing the two modalities. As a consequence, these studies commonly confirm that the global language control mechanisms involved independent processing between spoken production and written production, with unbalanced bilinguals exhibiting greater global language control in the dominant L1 when speaking and similar levels of global language control between L1 and L2 in writing.

### 4.2. Relationship between Bilingual Language Performance and Cognitive Control

The second research aim explored the relationship between the participants’ cognitive control and their language processing performance, and whether these effects were modulated by the types of production modalities.

The results revealed that participants with lower inhibitory control abilities (i.e., a greater Simon effect) had a larger magnitude of language dominance effect (i.e., larger naming speed difference between L2 and L1) in the single-language context in spoken and written modalities. In addition, we also found that the participants with stronger reactive control preferences (i.e., lower BSI score) tended to perform with a larger magnitude of the reversed language dominance effect (i.e., larger naming speed difference between L1 and L2) in the mixed-language context in spoken and written modalities. A previous study observed that better inhibitory control (which was measured using a go/no-go task) was correlated with larger reversed language dominance effects in a mixed-language context [43]. Another previous study explored the impact of bilinguals’ overall balance abilities between their dominant language and non-dominant language on the dominant language effect and the reversed dominant language effect, indicating that the lower-balanced bilinguals exhibited larger language dominance effects in a single-language context but not in a mixed-language context [68]. Combining the findings of this study and the two above-mentioned studies, it can be roughly inferred that bilinguals’ language balance abilities and cognitive control abilities influence their lexical accessibility between their dominant language and their non-dominant language.

Furthermore, we found that a lower Simon effect was associated with larger L2 mixing costs in spoken production but not in written production, which suggests that bilinguals solve global cross-language interference by inhibiting the non-dominant language during spoken production. This result was consistent with the findings of Jylkkä et al. [14] (in which inhibitory control was measured using the Flanker task). More specifically, bilinguals might engage in more inhibition in their weaker L2 to exert less global language control than in their dominant L1, leading to asymmetrical mixing costs across languages in spoken production. Inconsistent with prior studies, we did not find a significant association between inhibitory control and language switch costs. Notably, some previous studies have reported that inhibitory control is correlated with language switch costs [36,38,43,69], but the correlation trends are inconsistent (positive or negative). Some studies have reported that the Simon effect is negatively correlated with the L1 switch cost [43,69], while several other studies have demonstrated that the Simon effect or Flanker effect is positively correlated with L1 switch cost [36,38]. Another study has even reported that there is no correlation trend between the Simon effect and L1 switch cost [14]. Similar confounding findings have also been observed between the Simon effect and L2 switch costs [36,38,43,69]. The variable association between inhibitory control and local language control may stem from the dynamic nature of local language control processing, which may determine the specific involvement of cognitive control resources.

Finally, we also found that the BSI score significantly negatively predicted the mixing costs in both L1 and L2 in written production but not in spoken production, suggesting that the bilinguals with stronger proactive control were more likely to engage stronger in global language control when writing. Numerous previous studies have used AX-CPT to examine the association between cognitive control and bilingualism [46,47,48,51]. For example, Morales et al. [46] concluded that, relative to monolinguals, bilinguals can better coordinate proactive and reactive control to modulate cognitive control strategies. Subsequently, Bonfieni et al. [48] extended the results of Morales et al. [46] and found that bilinguals with higher proficiencies relied more on reactive control. Similarly, several studies have found that a greater language entropy, which reflected bilinguals’ overall balance (or diversity) of language usage across communicative contexts, was more reliant on proactive control [49,60]. These studies collectively suggest that bilinguals with different language proficiencies and complexities of language experience employ variable cognitive control strategies.

A study by Beatty-Martínez et al. [51] further explored how different bilingual experiences mediate the relationship between cognitive control and lexical access. They compared the performance of highly proficient bilinguals who lived in three different interaction contexts on a blocked language naming task and an AX-CPT task. The contexts of groups included separated bilingual contexts (in which individuals primarily use L1 in daily life and communication), integrated bilingual contexts (in which individuals use the two languages frequently and live in a predominantly L1 environment), and varied contexts (in which individuals live in a predominantly L2 language environment). The results from this study revealed that there was greater reliance on reactive control for separated context bilinguals and greater general reliance on proactive control for bilinguals with more mixed language experiences (integrated and varied bilingual contexts). In addition, this study also found association patterns for bilinguals in a separated context, in which greater a reliance on reactive control processes predicted a higher language naming accuracy in both L1 and L2. That is, there was an association between reactive control processes and bilingual language control for bilinguals in the separated context. In fact, the unbalanced bilinguals in present study who used L1 as their predominant daily language also belonged to the group of bilinguals with separated contexts. In the present study, one potential explanation for the association between the proactive control preference and mixing costs is that there is globally induced cross-language competition in bilingual production. The global control balance between the dominant language and non-dominant language in written production relies more on cognitive control strategy regulation.

### 4.3. Limitations

The present study has several potential limitations. Firstly, the sample in this study was gender imbalanced, with more than two-thirds of the participants being female. This study did not include gender in the analysis, thereby ignoring the potential influence of gender. Secondly, self-assessed language proficiency, while of some valuable, may have limitations in terms of reliability and objectivity, potentially differing slightly from participants’ actual language proficiency levels. However, when combined with the results of the second language proficiency test (lexTALE test; Lemhofer and Broersma, [54]), we could confirm that the participants in this study can be categorized as unbalanced bilinguals. In future research, using more rigorous and objective tools to assess multiple language proficiencies may provide a more accurate representation of the participants’ actual language proficiencies. Finally, there is a potential limitation of color squares as language cues, which may involve some additional processing beyond language control. Participants need to first convert the cue into the language labels and then associate the language labels with the target stimuli. Therefore, in the future, more direct language cues or other better cue-to-language mapping methods can be considered to further explore the pure language control effect.

## 5. Conclusions

In conclusion, the present study explores whether the subcomponents of language control induced from different production modalities were regulated by individual variability in cognitive control. We found similar processing patterns in local language control and different processing patterns in global language control between spoken production and written production for unbalanced bilinguals. Specifically, there were symmetrical switch costs across languages in both modalities, while there was a reversed language dominance effect and asymmetrical mixing costs in spoken production but not in written production. In addition, we also found that the Simon effect negatively predicted the L2 mixing costs in spoken production, while BSI negatively predicted the L1 mixing cost and L2 mixing costs in written production. These indicated that different aspects of cognitive control modulated global language control of the two modalities. The findings of this study provide new insights into the association between individuals’ cognitive control and bilingual language control.

## Figures and Tables

**Table 1:** Participant characteristics in the present study (standard deviations in parentheses).

	L1 (Chinese)	L2 (English)	*t*	*p*
Self-rating proficiency				
Listening	6.35 (0.70)	3.72 (1.01)	18.59	<0.001
Speaking	6.16 (0.85)	3.58 (1.03)	17.01	<0.001
Reading	6.06 (0.79)	4.34 (0.92)	13.03	<0.001
Writing	5.23 (0.97)	3.81 (0.88)	9.58	<0.001
L2_AoA		8.45 (2.35)		
LexTALE		54.21 (8.09)		

**Table 2:** Descriptive results of the mean latencies and error rates for the spoken and written modalities.

		Spoken Modality		Written Modality	
		L1	L2	L1	L2
Latencies (ms)					
	Switch	875 (202)	833 (192)	1117 (281)	1101 (281)
	Repeat	805 (192)	773 (173)	1037 (261)	1014 (259)
	Single	669 (132)	682 (119)	893 (196)	886 (185)
	Switch costs	69	60	79	87
	Mixing costs	136	92	147	132
Error rates (%)					
	Switch	5.2 (5.9)	2.8 (2.7)	3.0 (3.3)	3.4 (3.9)
	Repeat	3.3 (4.5)	1.2 (2.0)	1.8 (2.4)	2.4 (2.8)
	Single	0.2 (0.8)	0.3 (1.0)	0.2 (0.7)	0.4 (0.9)
	Switch costs	1.9	1.7	1.2	1.0
	Mixing costs	3.0	0.8	1.7	2.0

**Table 3:** Results of LME examining the switch costs and mixing costs separately between spoken and written modalities for log latency data.

Fixed Effects	Estimate	*SE*	*t* Value	*p*
Switch costs				
Intercept	6.815	0.017	404.30	**<0.001**
Modality	0.260	0.016	16.12	**<0.001**
Language	0.029	0.013	2.27	**0.039**
Trial type (repeat vs. switch)	0.077	0.007	11.35	**<0.001**
Modality × Language	−0.022	0.007	−3.20	**0.002**
Modality × Trial type	0.000	0.009	0.01	0.990
Language × Trial type	0.000	0.006	0.01	0.993
Modality × Language × Trial type	−0.018	0.011	−1.56	0.119
Mixing costs				
Intercept	6.705	0.015	458.78	**<0.001**
Modality	0.265	0.016	16.21	**<0.001**
Language	0.010	0.011	0.90	0.380
Trial type (repeat vs. single)	−0.143	0.008	−17.14	**<0.001**
Modality × Language	0.007	0.012	0.59	0.559
Modality × Trial type	0.010	0.009	1.16	0.252
Language × Trial type	−0.037	0.008	−4.48	**<0.001**
Modality × Language × Trial type	0.041	0.013	3.11	**0.003**

**Figure A1:**
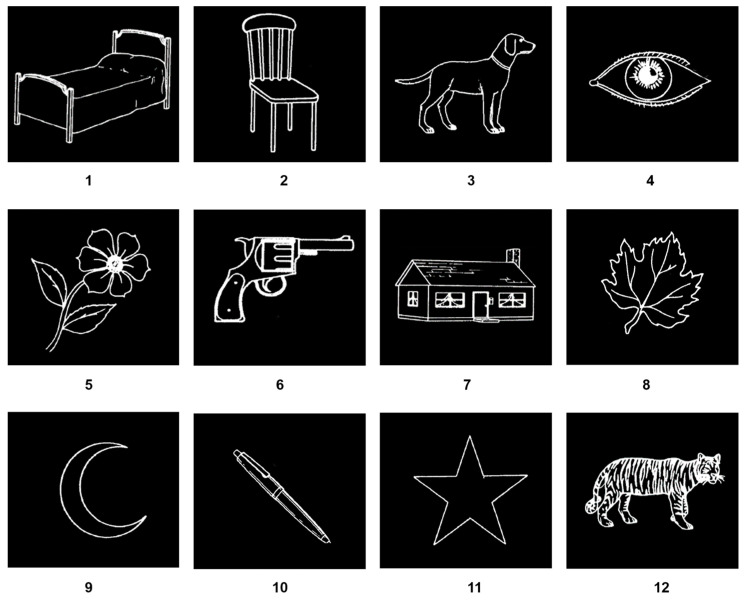
Experimental pictures used in the cued language switching task. Note: The number below each picture represents the item number, which corresponds to the Chinese and English names in Table A1.

## Data Availability

The data that support the findings of this study are available from the corresponding author, Wang, R upon request. The data are not publicly available because they contain information that could compromise the privacy of the research participants.

## Appendix A

**Table A1:** Experimental stimuli used in the cued language switching task.

	English Name	Chinese Name
1	bed	床
2	chair	椅
3	dog	狗
4	eye	眼
5	flower	花
6	gun	枪
7	house	房
8	leaf	叶
9	moon	月
10	pen	笔
11	star	星
12	tiger	虎
